# Awareness and associated factors of prepackaged food nutrition labels by college students in China: a cross-sectional study

**DOI:** 10.3389/fpubh.2025.1577633

**Published:** 2025-06-26

**Authors:** Qian Liu, Jinyi Chen

**Affiliations:** Department of Food Nutrition and Safety, Sanda University, Shanghai, China

**Keywords:** food nutrition label, prepackaged food, college student, food nutrition courses, cross-sectional study

## Abstract

**Background:**

The effective utilization of nutrition labels on prepackaged foods can facilitate the cultivation of healthful dietary behaviors among college students, thereby mitigating the risk of chronic non-communicable diseases. The study assessed the ability of college students in China’s economically and educationally highly developed provinces to use food nutrition labels and explored the associated factors affecting the awareness of food nutrition labels.

**Methods:**

A crosssectional study was conducted among college students enrolled in five higher education institutions in China. This study used a structured questionnaire method to collect data, descriptive statistics, and multivariate logistic regression to analyze and process the data.

**Results:**

71.8% of college students had low awareness of food nutrition labeling. Awareness of food nutrition labeling was positively correlated with the degree of need (OR: 1.903; 95% Cl: 1.513–2.394), frequency of use (OR: 1.780; 95% Cl: 1.537–2.062), and trust (OR: 1.113; 95% Cl: 1.009–1.229), and was negatively correlated with the study of nutrition courses (OR: 0.655; 95% Cl: 0.528–0.812). The most important way for college students to acquire knowledge about food nutrition labeling was online videos (52.4%, *n* = 1,318). Furthermore, frequent utilization of nutritional labels in food choices, high demand, and high trust in food nutrition labels play a significant role in enhancing the awareness among college students in economically and educationally highly developed provinces.

**Conclusion:**

In light of our finding, we propose a reform of the existing nutrition education system to enhance nutritional awareness among college students through a more diversified set of instructional approaches and the implementation of compulsory nutrition education at an earlier stage. Leveraging popular media platforms to widely distribute professing nutritional information during daily public nutrition education is also advised.

## 1 Introduction

The current national fatality rate from chronic diseases stands at 71%, escalating to 88.5% within China, posing a significant public health concern on a global scale (1). Obesity emerges as a predominant risk factor for chronic ailments (2), with projections indicating a rise to 65.3% among obese Chinese adults by 2030 (3). Notably, chronic diseases like obesity exhibit a strong correlation with poor dietary habits (4), contributing to a spectrum of medical conditions (5–8). In order to prevent the incidence of chronic diseases, each country is committed to developing effective public strategies to protect national health, including the implementation of food nutrition labeling aimed at guiding consumers to make rational food choices to reduce the risk of chronic non-communicable diseases. In daily life, 71% of consumers reported that they would check the nutrition label of prepackaged food and obtain nutritional information before purchase (9, 10). According to existing studies, consumers who regularly use food nutrition labels have healthier diets (11, 12). However, young consumers are susceptible to the influence of the commodity environment and thus choose unhealthy foods (13). And college students are the main group of young consumers. Therefore, the increasing prevalence of chronic diseases globally highlights the importance of understanding nutrition labels, particularly among young adults who are forming lifelong dietary habits.

The primary method for gaining insight into food involves analyzing the nutritional data on packaged food. According to China’s current prepackaged nutrition labeling standers GB7718-2011 and GB28050-2011 (14, 15) prepackaged foods for direct purchase by consumers are required to be labeled with nutrition labels (excluding foods exempt from labeling). These nutrition labels are required to encompass the following elements: Nutritional Composition Table, which consists of three columns detailing nutrient names, content values, and nutrient reference value percentages (NRV%); The “1 + 4” core nutrients, including energy, protein, fat, carbohydrates, and sodium, must be specified on the labels of all prepackaged foods (with exceptions noted), along with their respective percentages of the NRV%. Nutrition labeling provides consumers with relevant descriptions of the nutritional information and characteristics of foods to help them understand the food and promote healthy dietary behaviors.

For college students, food nutrition-related courses or nutrition interventions can improve the ability to analyze food nutrition labels and cultivate positive food nutrition label reading habits. In China, nutrition courses typically cover fundamental nutrition concepts such as the evolution of nutrition, basic principles, and recommended dietary intake for Chinese individuals.

While previous studies have examined consumer behavior regarding nutrition labels, limited research explores the awareness and influencing factors among university students in developing countries. And, the relationship between nutrition curriculum education and nutrition labeling awareness is uncertain. Moreover, the factors impacting college students’ awareness of nutritional labeling in well-developed regions remain unclear. Globally, balanced nutritional intake is rarely considered in national public health agendas, especially in early developing countries. Balanced dietary intake should be regarded as an important pillar of health, and in order to improve the balance of nutritional intake in developing countries, attention needs to be paid to education and awareness, research, and targeted public health policies (16). Existing studies have shown that health inequalities in developing countries are exacerbated by factors such as socioeconomic level and education (17, 18), and such inequalities are more prominent between rural and urban areas in developing countries (19). In China, the world’s second-largest economy and the largest developing country, nutrition science and practice have changed significantly over the past decade, with national nutritional needs shifting from the prevention of nutritional deficiencies to the fulfillment of dietary nutrient requirements for optimal health and planetary sustainability. However, most of the existing research on food nutrition labeling in the Chinese university student population focuses on underdeveloped provinces of China, where there is a significant imbalance in socio-economic development, quality of nutritional education, and level of nutritional literacy between the underdeveloped and developed provinces of China.

Therefore, this study aims to analyze the awareness and use of prepackaged nutrition labels among the college student population in economically developed provinces in China and identify the influencing factors that affect nutrition label awareness and use among this population in economically developed provinces. Exploring the effectiveness of nutrition education policy in economically educated provinces in terms of awareness and use of nutrition labels and to help government officials optimize the current labeling regulations. As these programs are complex sustainable development issues that are linked to health through nutritional intake but also to sustainable economic development, the environment, and trade, in a broader context, insights can be provided to other developing countries for nutritional public health policy development and nutrition curriculum interventions.

## 2 Materials and methods

### 2.1 Research design and settings

This study will describe the awareness of prepackaged nutrition labels among college students in economically developed provinces in China, identify the factors that influence this group’s awareness of nutrition labels in economically developed provinces, and explore the impact of nutrition education programs in economically developed provinces on awareness of nutrition labels. Understanding these factors can inform interventions through governmental policies, the dissemination of nutrition labeling knowledge, and the enhancement of nutrition label presentation to facilitate chronic disease prevention and the development of sustainable nutrition education programs. Specifically, the following hypotheses were tested:

1. There is a positive correlation between the frequency of nutrition label use among college students and their awareness of nutrition labels.
2. The degree of trust that college students place in nutrition labels positively influences their nutritional awareness.
3. There is a positive correlation between the demand for nutrition labels and college students’ awareness of nutrition labels.
4. The enrollment of college students in food nutrition courses does not exert a positive influence on their awareness of nutrition label.

### 2.2 Research design and settings

This study used a cross-sectional design to examine college students’ awareness of food nutrition labeling and the factors that influence it, as well as whether their study of nutrition courses and access to food nutrition information have an impact on awareness. Simple random sampling was used, and 5 universities offering full-time undergraduate education in Shanghai, China, were sampled. Participants had full-time student status. All subjects signed an informed consent form. All information was collected by a questionnaire. The survey included basic demographic information (students’ gender, grade, height, weight, and major of study), whether they studied nutrition courses, use of nutrition labels, and nutrition labeling knowledge test.

### 2.3 Instrumentations

In this study, we reviewed and sorted out the existing domestic and international literature on nutritional labeling research on Chinese college students to have a theoretical grasp of nutritional labeling research on Chinese college students. On this basis, the questionnaire for assessing the awareness of prepackaged nutrition labeling among college students in Shanghai, China, was integrated and designed based on the General Principles of Nutrition Labeling of Prepackaged Foods (GB28050-2011, GB7718-2011) and the Nutritional Pagoda of Dietary Nutrition for Chinese Residents, with the questionnaire by Wei et al. (20) as a reference to ensure the validity of the questionnaire. After formulating the questionnaire, its content validity was assessed using an expert assessment method (21). A total of six experts, including experts in the fields of nutrition, public health, food safety, and epidemiology, were invited to review the content validity of the questionnaire. The outcomes of the expert appraisal are delineated in Table 1.

**Table 1:** Content validity expert scores and content validity index calculation.

Part	Expert ratings						Number of experts rated 3 or 4	I-CVI	Pc	K*	K* value evaluations
	A	B	C	D	E	F					
A											
1	4	4	4	4	3	3	6	1	0.016	1	Excellence
2	3	3	3	3	3	3	6	1	0.016	1	Excellence
3	3	3	3	3	2	3	5	0.833	0.094	0.816	Excellence
4	3	3	3	3	2	3	5	0.833	0.094	0.816	Excellence
5	3	4	4	3	3	3	6	1	0.016	1	Excellence
6	3	4	3	4	3	4	6	1	0.016	1	Excellence
B											
1	3	3	3	3	3	4	6	1	0.016	1	Excellence
2	3	4	2	4	3	3	5	0.833	0.094	0.816	Excellence
3	3	4	3	3	3	2	5	0.833	0.094	0.816	Excellence
4	3	3	4	2	3	4	5	0.833	0.094	0.816	Excellence
5	3	3	4	3	3	4	6	1	0.016	1	Excellence
6	3	3	3	3	3	3	6	1	0.016	1	Excellence
C											
1	3	3	3	3	3	4	6	1	0.016	1	Excellence
2	3	4	4	3	3	3	6	1	0.016	1	Excellence
3	3	3	3	3	4	3	6	1	0.016	1	Excellence
4	4	3	2	4	4	3	5	0.833	0.094	0.816	Excellence
5	3	3	4	3	2	3	5	0.833	0.094	0.816	Excellence
6	3	3	4	3	3	3	6	1	0.016	1	Excellence
7	3	3	3	3	2	4	5	0.833	0.094	0.816	Excellence
8	4	2	3	3	4	4	5	0.833	0.094	0.816	Excellence
9	3	4	3	3	3	4	6	1	0.016	1	Excellence
10	4	3	2	3	3	4	5	0.833	0.094	0.816	Excellence
11	3	3	4	3	2	3	5	0.833	0.094	0.816	Excellence
12	2	2	3	3	2	3	3	0.5	0.313	0.273	Poor

“*” is not a stand-alone symbol; K* is an overall symbol that refers to correcting content validity (adjusting the kappa value), which is usually labeled as K*.

Since the I-CVI of question 12 = 0.5, which was approved by only 3 experts, we removed question 12 from the questionnaire. After the removal, the calculation of the mean of each I-CVI resulted in a S-CVI/Ave of 0.917 > 0.900. Considering all the factors, the questionnaire’s content validity was acceptable. After the expert review, we first conducted a pre-survey on 100 undergraduate students whose majors were not related to food science, using stratified sampling. This was done to test the readability of the questionnaire and to determine the average time required to complete it, which served as a reference for excluding abnormal responses. The 2,517 questionnaires that met the inclusion criteria were analyzed for internal consistency, expressed as Cronbach’s alpha coefficient *α* = 0.721 and Barlett’s sphericity test (*X*^2^ = 3030.316, *p* < 0.001).

The questionnaire includes three parts: basic information, use of nutrition labeling, and nutrition labeling knowledge test, of which the basic information includes six questions: A1-gender, A2-height, A3-weight, A4-professional, A5-grade level and A-6 whether you have taken nutrition courses.

The use of nutrition labels includes: B1-Do you use nutrition labels when you buy food? (FUNL), B2-What information do you focus on when you use food labels? B3-What influences you to use nutrition labels? B4-What media channels do you often use to obtain nutrition-related knowledge? B5-How much do you trust food nutrition labels? (TNL), B6-Do you think that the presence of nutrition labels is necessary in your daily food purchases (DNL)? Total 6 questions.

The nutrition labeling knowledge test consisted of a total of 11 questions and they were all single-choice questions with each question carrying 2 points out of a total of 22 points. The content included: C1-What do vegetables mainly provide?, C2-What is the recommended daily salt intake for Chinese residents?, C3-What is the meaning of “Recommended National Dietary Nutrient Intake, C4-What does “sugar-free” mean on food labels?, C5-Which of the following is a mandatory symbol for food nutrition labeling?, C6-What is the meaning of NRV% in the following images?, C7-How much of an adult’s total energy intake for the day should be carbohydrates?, C8-Daily dietary fat should provide no more than 30% of total energy?, C9-Are the following foods suitable for children?, C10-Nutrients that must be labeled on nutrition labels include: protein, fat, carbohydrates, and sodium? and C11-Does food labeling refer to words, graphics, symbols, and descriptions on food packaging?. Referring to the cognitive scoring rules of similar studies (20), we categorized the level of awareness as low (score < 60% of full score), medium (60% ≤ total score < 80% of full score), and good (total score ≥ 80% of full score) based on the total score of the knowledge test.

### 2.4 Ethical consideration and data collection

This study was conducted according to the guidelines in the Declaration of Helsinki. The Ethics Committee of Shanghai Sanda College approved all procedures involving human subjects (ID number: 2024005). To enhance the credibility and precision of the survey instrument, 10 researchers were enlisted from each university. A designated team leader was appointed at each university, overseeing the training of team members over a two-day period. This training encompassed elucidating the study’s significance and objectives, elucidating the content of each questionnaire item, emphasizing data quality, specifying daily sample size requirements, detailing data collection and organization procedures, and addressing safety protocols.

After the training, the teams conducted a real-time offline electronic questionnaire survey from September 24 to 27, 2024, using the professional online research software “Questionnaire Star” (22) among the university students who met the inclusion criteria. The research subjects were informed and voluntarily participated in the study. The research subjects completed the questionnaire, with the first page providing unified guidance on describing terms. The respondents independently completed the questionnaire. 2,545 college students participated in the study, and 2,517 questionnaires were included (response rate of 98.8%) after the researchers had eliminated invalid samples (Questionnaire completion time < 1 min).

### 2.5 Statistical analysis

SPSS 23.0 was used for analysis. Descriptive statistical analysis was utilized for primary data, frequency, and percentage were used for count variables, and comparisons between groups were made using the chi-square test. To explore whether there is a relationship between different nutrition courses of study and basic demographic information and their awareness of prepackaged nutrition labels. The student’s awareness level served as the dependent variable, while gender, study nutrition courses, FUNL, TNL, and DNL (a total of 5 items) were utilized as independent variables. Logistic regression was used to assess the impact of students’ basic information and usage of prepackaged food nutrition labels on their awareness. Statistical significance was indicated by *p* < 0.05 and *p* < 0.001.

## 3 Results

### 3.1 Basic information about the respondents

A total of 2,517 students were included in the study, comprising 716 males (28.4%) and 1801 females (71.6%). The distribution across different grades was as follows: 1,698 freshmen (67.5%), 393 sophomores (15.6%), 355 juniors (14.1%), and 71 seniors (2.8%). Among the participants, 1,640 (65.2%) students attended colleges offering nutrition courses, while 1853 (73.6%) reported not having taken a nutrient related course. The characteristics of the basic demographics of college students are in Table 2.

**Table 2:** Basic demographics of college students in China, September 2024.

Characteristic	*n*	%
Gender		
Male	716	28.4
Female	1801	71.6
Grade		
Freshman	1,698	67.5
Sophomore	393	15.6
Junior	355	14.1
Senior	71	2.8
College		
Business^1^	418	16.6
Law	102	4.1
Medical^1^	299	11.9
Management^1^	675	26.8
Engineering	225	8.9
Education^1^	248	9.9
Languages	134	5.3
Information Technology	149	5.9
Art design	267	10.6
Study nutrition course		
Yes	664	26.4
No	1853	73.6

1 Nutrition courses are offered at the college.

#### 3.1.1 Nutrition labels knowledge exam on prepackaged nutrition labeling

Overall, the error rates of students who had taken the nutrition course were all lower than those of students who had not taken the nutrition course. In five topics, C1, C2, C4, C8, and C10, there was a highly significant difference (*p* < 0.001) between the studied and non-studied the nutrition course groups. Secondly, the topics with high error rates were C6-What is the NRV% meaning of the following images? accounting for 61.7%, C5-Which of the following is a mandatory mark for nutrition labeling of food? accounting for 59.4%, and C3-What does “Recommended National Dietary Nutrient Intakes” mean? accounting for 59.3%, and there was no significant difference between the groups for the above high error rate topics (*p* > 0.05). The error rates of students’ responses in different learning situations are shown in Table 3.

**Table 3:** Error rates of students with different learning characteristics.

Question	Total	Study nutrition course		*p-*value
		Yes	No	
	*n* = (2517)	*n* = (664)	*n* = (1853)	
C6-What is the NRV% meaning of the following images?	1,553 (61.7%)	392 (25.2%)	1,161 (74.8%)	0.100
C5-Which of the following is a mandatory mark for nutrition labeling of food?	1,497 (59.4%)	388 (25.9%)	1,109 (74.1%)	0.524
C3-What does “Recommended National Dietary Nutrient Intakes” mean?	1,495 (59.3%)	387 (25.9%)	1,108 (74.1%)	0.496
C10-Core nutrients for nutrition labeling include: protein, fat, carbohydrates and sodium?	1,059 (42.0%)	377 (35.6%)	682 (64.4%)	<0.001
C2-Recommended daily salt content in China	1,027 (40.8%)	319 (31.1%)	708 (68.9%)	<0.001
C4-What does “sugar-free” mean in food labeling?	1,006 (39.9%)	310 (30.8%)	696 (69.2%)	<0.001
C8-Daily dietary fat should not provide more than 30% of total energy?	999 (39.6%)	327 (32.7%)	672 (67.3%)	<0.001
C7-Dietary carbohydrates should account for energy?	899 (35.7%)	218 (24.2%)	681 (75.8%)	0.070
C11-Does food labeling refer to the words, graphics, symbols and descriptions on food packaging?	887 (35.2%)	255 (28.7%)	632 (71.3%)	0.047
C1-What are the main things that vegetables provide you with?	779 (31.7%)	269 (34.5%)	510 (65.5%)	<0.001
C9-Are the following foods suitable for children?	568 (22.5%)	160 (28.2%)	408 (71.8%)	0.272

Yes means: Questionnaire responses to having studied a food nutrition course. No means: Questionnaire responses did not study a food nutrition course.

### 3.2 Analysis of factors influencing awareness

#### 3.2.1 Single-factor analysis of awareness

In the examination of the level of awareness regarding nutrition labels on prepackaged food items, it was found that a low level of awareness of nutrition labels of prepackaged foods in 71.8% (*n* = 1807) of the students, a medium level of awareness in 27.9% (*n* = 702) of the students, and a good level of awareness in 0.3% (*n* = 8) of the students. There were significant variations among groups in 4 independent variables: FUNL, TNL, DNL, and whether they had studied nutrition education (*p* < 0.001). Furthermore, there was no statistical significance in the independent variables of gender and whether nutrition courses were offered (*p* > 0.001). Single factor analysis of awareness in Table 4.

**Table 4:** A one-factor analysis of student awareness.

Factor	Total	Degree 1^1^	Degree 2^2^	Degree 3^3^	*χ^2^*	*p*-value
	(*n* = 2,517)	(*n* = 1807)	(*n* = 702)	(*n* = 8)		
Gender					8.775	0.012
Male	716 (28.4%)	543 (30.0%)	170 (23.7%)	3 (37.5%)		
Female	1801 (71.6%)	1,264 (70.0%)	532 (75.8%)	5 (62.5%)		
FUNL					83.081	<0.001
Never	334 (13.2%)	301 (16.6%)	33 (4.7%)	0 (0.0%)		
Sometimes	1,421 (56.5%)	1,024 (56.7%)	392 (55.8%)	5 (62.5%)		
Usual	762 (30.3%)	482 (26.7%)	277 (39.5%)	3 (37.5%)		
TNL					54.613	<0.001
Very distrustful	145 (5.8%)	129 (7.1%)	16 (2.3%)	0 (0.0%)		
Distrust	162 (6.4%)	140 (7.7%)	22 (3.1%)	0 (0.0%)		
Moderate trust	1,309 (52.0%)	907 (50.2%)	399 (56.8%)	3 (37.5%)		
Trust	634 (25.2%)	429 (23.7%)	200 (28.5%)	5 (62.5%)		
Very trustful	267 (10.6%)	202 (11.2%)	65 (9.3%)	0 (0.0%)		
DNL					50.636	<0.001
Necessarily	1994 (79.2%)	1,373 (76.0%)	613 (87.3%)	8 (100%)		
Normal	457 (18.2%)	369 (20.4%)	88 (12.5%)	0 (0.0%)		
Unnecessarily	66 (2.6%)	65 (3.6%)	1 (0.1%)	0 (0.0%)		
Offer nutrition course					4.490	0.106
Yes	1,640 (65.2%)	1,115 (63.9%)	479 (68.2%)	6 (75.0%)		
No	877 (34.8%)	652 (36.1%)	223 (31.8%)	2 (25.0%)		
Study nutrition course					22.621	<0.001
Yes	664 (26.4%)	520 (28.8%)	140 (19.9%)	4 (50%)		
No	1853 (73.6%)	1,287 (71.2%)	562 (80.1%)	4 (50%)		

^1^Degree 1 means low level of awareness, ^2^Degree 2 means medium level of awareness, ^3^Degree 3 means good level of awareness. The chi-square (X^2^) was used to test the differences between different groups.

#### 3.2.2 Multifactorial analysis of student awareness

The univariate regression analysis did not account for the overall influence of other variables, and only those univariate variables with significant indicators were included in the sequential multifactor logistic regression analysis. The dependent variables were categorized as follows: 1 = low recognition, 2 = medium recognition, 3 = good recognition. The definitions and assignments of independent variables were as follows: Nutrition courses study: 1 = no, 2 = yes; FUNL: 1 = never, 2 = sometimes, 3 = usually; TNL: 1 = very distrustful, 2 = distrustful, 3 = moderate trust, 4 = trust, 5 = very trustful; DNL: 1 = unnecessary, 2 = normal, 3 = necessary. A significance level of *p* < 0.05 indicated statistical significance and *p* < 0.001 indicated extreme significance. The results of the regression analysis are presented in Table 5.

**Table 5:** Ordered logistic regression analysis of student awareness and its correlates.

Factor	*β*	SE	Wald χ2	*p*	OR	95% CI
Study nutrition course	−0.423	0.110	14.913	<0.001	0.655	0.528, 0.812
Frequency of use of prepackaged nutrition labels	0.577	0.075	59.214	<0.001	1.780	1.537, 2.062
Trust in nutrition labels of prepackaged foods	0.107	0.050	4.579	<0.050	1.113	1.009, 1.229
Demand for nutrition labels of prepackaged foods	0.644	0.117	30.212	<0.001	1.903	1.513, 2.394

According to the results of the regression analysis, the frequency of prepackaged food nutrition label utilization (OR = 1.780, CI: 1.537, 2.062), trust in prepackaged food nutrition labels (OR = 1.113, CI: 1.009, 1.229), and the demand for prepackaged food nutrition labels (OR = 0.530, CI: 0.419, 0.667) had a substantial influence on awareness (*p* < 0.001). Specifically, when the frequency of prepackaged food nutrition label use, the trust level in prepackaged food nutrition labels, and the need for prepackaged food nutrition labels increased, the awareness of nutrition labels also increased by factors of 1.780, 1.113, and 1.903, respectively. However, it is noteworthy that whether or not participants had studied a nutrition course (OR = 0.655, CI: 0.528, 0.812) had a significant impact on awareness (*p* < 0.05), indicating that even if individuals had received nutrition education, their awareness of related knowledge was not necessarily improved.

### 3.3 Use of nutrition labels of prepackaged foods for students

#### 3.3.1 Focus on content and frequency of use

**Figure 1:**
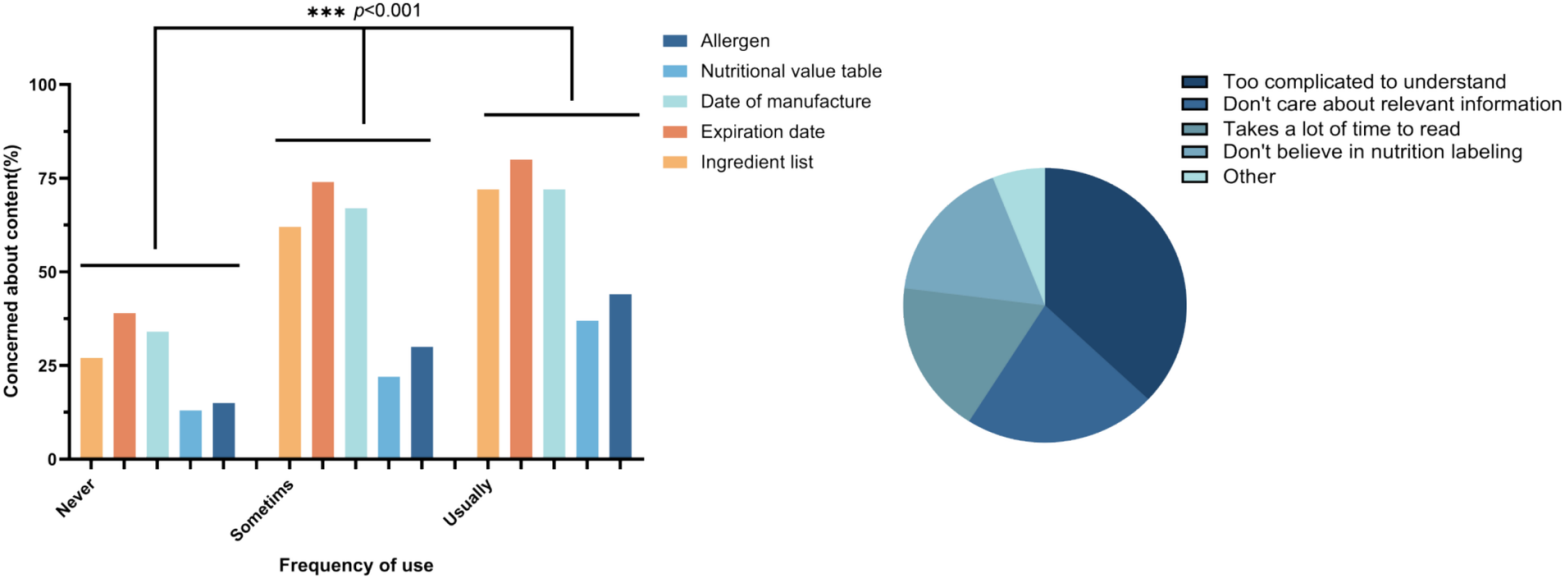
Student concerns and troublesome usage factors for different frequencies of use.

Using a chi-square test between groups with different frequencies of use of nutrition labels, we found that the content of students’ concerns about nutrition labels on prepackaged foods differed from their frequency of use. The majority of students pay the most attention to the shelf-life label (71.6%), followed by the production date (66.6%) and the ingredient list (62.4%) for those with medium frequency of use. Students with the highest frequency of use pay more attention to allergens (41.2%). There were significant differences among the different frequency groups (*p* < 0.001). When it comes to the use of prepackaged nutrition labels, 36% of students find them too complicated to understand, 22% do not care about the information, and 17% do not believe in nutrition labels. The specific concerns across different frequency groups and factors preventing students from using labels are both depicted in Figure 1.

#### 3.3.2 Analysis of students’ awareness and ways of acquiring nutritional knowledge

**Figure 2:**
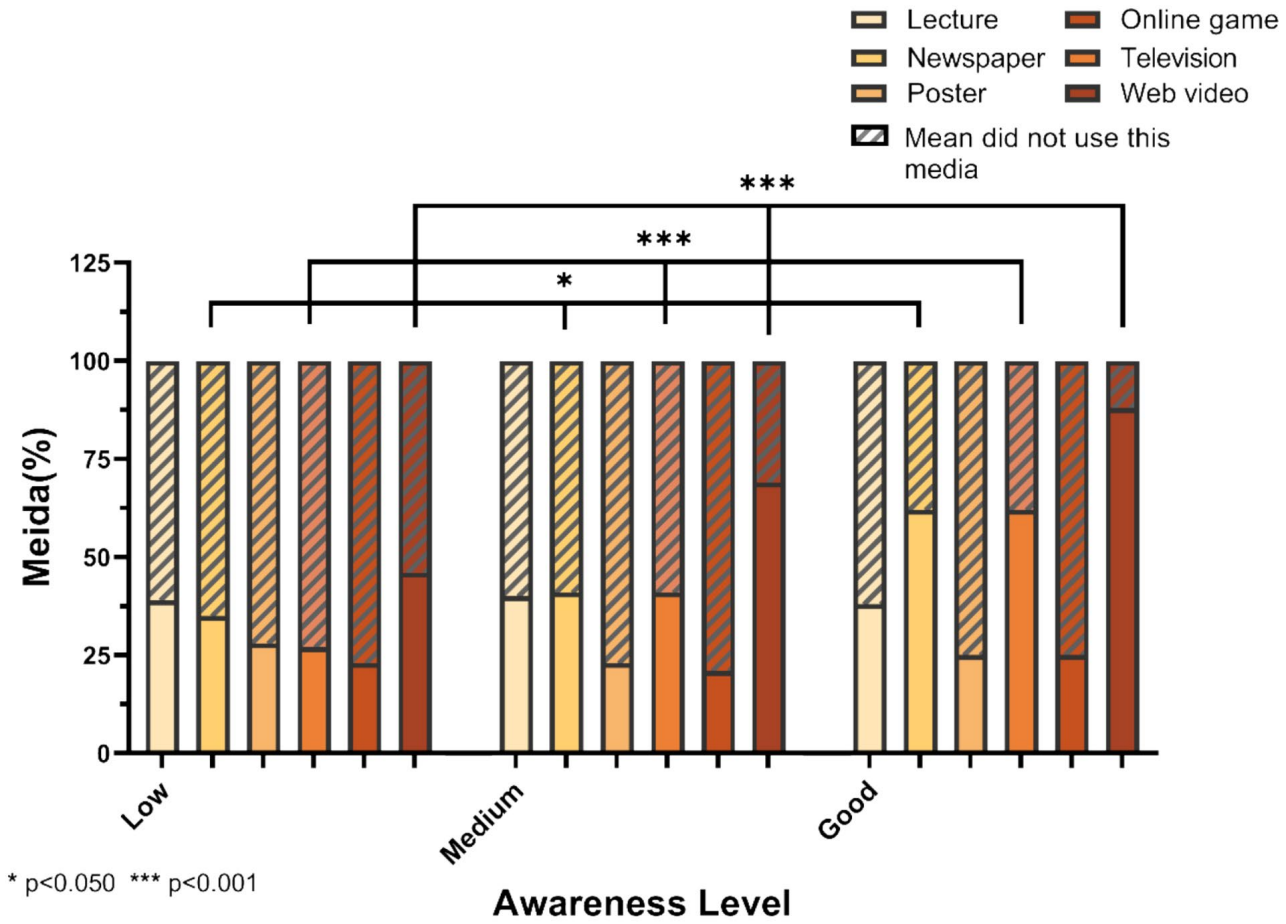
Different levels of media use and awareness.

A total of 1,318 college students (52.4%) mainly acquired knowledge about nutrition labels through web videos. Relatively few people gained knowledge through lectures (39.4%), posters (26.5%), and online games (22.4%). Among them, students with low awareness mainly acquired knowledge through web videos, accounting for 45.8%. The method of knowledge acquisition through newspapers and periodicals showed significant differences compared to those who did not acquire knowledge through newspapers and periodicals (*p* < 0.05). There were also significant differences between those who acquired knowledge through TV, web videos compared to those who did not (*p* < 0.001). Additionally, there was no significant difference in cognition among the knowledge groups who acquired relevant knowledge through lectures, posters, and online games (*p* > 0.001). The differences in awareness of nutrition labels on prepackaged foods through various methods of acquiring prepackaged nutrition labels are outlined in Figure 2.

## 4 Discussion

This study conducted a cross-sectional survey to assess college students’ knowledge and understanding of nutrition labeling for prepackaged food in China. The objective of this study was to evaluate the comprehension and practical usage of prepackaged food nutrition labels among college students in China, as well as the incorporation of relevant food nutrition education in universities. Additionally, the study aimed to analyze the factors influencing awareness of food nutrition labels and from there we can find ways to raise awareness of prepackaged nutrition labeling among youth as a way to reduce obesity rates.

The findings revealed that college students in China exhibited limited understanding and application of prepackaged food nutrition labels, with 71.7% displaying low awareness and only 30.3% indicating frequent use of nutrition labels in their daily lives. A study in Xinjiang, China, in 2015 illustrated that 29.3% of Chinese students regularly read nutrition labels (23). Similarly, research in Malaysia found that approximately 37.6% of educated students have low awareness, and 53.6% of university students do not utilize nutrition labels (24). Moreover, a study in Saudi Arabia reported that 58.0% of consumers value reading nutrition labels (25), while the British consumer nutrition knowledge study indicated that 87.0% of consumers effectively use nutrition labels (26). In summary, despite improvements in the understanding of prepackaged food nutrition labels among Chinese college students after a decade of widespread utilization, they still exhibit lower awareness compared to countries that implemented labeling earlier (27). Some studies suggest that this performance may be associated with the disparity in nutrition knowledge between urban and rural areas (28, 29).

In our research, we discovered that 65.2% of the participants were from colleges offering nutrition courses, while only 26.4% had studied a nutrition course. This indicates that the current quality of nutrition education in China is inadequate, consistent with recent findings (30). The formation of the aforementioned issue can be attributed to several factors. First, nutrition education is not included in the study of different majors. In surveying the syllabi developed by the sampled higher education institutions, we found that these institutions did not offer nutrition courses (either optional or compulsory) in every major, a policy that leaves the university student population with an insufficient stock of nutritional knowledge (31). Whereas, nutrition education has a positive impact on college students’ use of nutrition labels and access to healthier diets (32). Students in majors where nutrition knowledge is not mandatory should increase their knowledge of specific nutrition areas based on existing problems (33) to achieve health intervention outcomes. In contrast, students pursuing careers that necessitate frequent utilization of nutritional knowledge, such as medical students, more specialized nutritional education should be mandatorily included in medical training to enhance their knowledge and apply it to clinical practice (34, 35). Although it is challenging to disseminate accurate nutrition knowledge to populations from diverse backgrounds, existing interactive online courses can be an effective means to facilitate substantive study (36). Second, education in nutrition programs is received too late. Due to economic and educational resource constraints, exposure to professional and systematic nutrition education in China is only possible in adulthood, and it is not mandatory. This design of education makes students miss out on the critical period of forming healthy eating habits—childhood and adolescence (37). In the United States, where nutrition education began earlier, independent nutrition education programs such as EFNEP and CSHP have been implemented for preschoolers and primary and secondary school students. Countries such as Japan and the United Kingdom have introduced corresponding laws to ensure that children’s nutrition education is carried out; for example, the School Lunch Law introduced in Japan in 1954 and the Education Reform Act of the United Kingdom in 1988, which incorporated nutrition courses into the compulsory curriculum for primary and secondary school students (38). Thirdly, there is a single form of instruction, and its content is easy. In the absence of the foundation of basic nutrition education in childhood, nutrition education in universities can only achieve the purpose of understanding basic nutrition knowledge. And there are no more ways for students to learn nutrition knowledge in China than through mandatory study in the curriculum, which makes it difficult for students to effectively apply that knowledge to promote healthy growth (39). However, in the United States, in addition to being included in all core and elective subjects or offered as a stand-alone course at all grade levels, curricular nutrition education also integrates nutritional knowledge with participatory activities in daily life through a variety of indirect means, such as taste tests and culinary education (40). This strategy has effectively enhanced the distribution of nutritional information beyond traditional educational settings, resulting in a substantial enhancement of students’ understanding of nutrition. Moreover, the establishment of the GENIUS network in colleges and universities across the United Kingdom serves to bolster the formulation of public health strategies geared toward fostering healthy dietary habits among students by connecting them with current food policies and expert knowledge in the field (41). Although the formulation of public policies is influenced by various factors, such as the economic status of a nation, advancements in technology have provided more convenient and cost-effective avenues for widespread nutrition education (42). Our research revealed that online videos were the primary source for extracurricular nutritional knowledge, and there were significant differences in the level of nutritional awareness among those who acquired nutritional knowledge through different methods. We suggested that the implementation of a comprehensive online nutrition course system could facilitate the daily dissemination of nutritional information on a large scale (43). Moreover, we also propose the prompt establishment of a tailored strategy for children’s nutrition education that aligns with the specific needs of China (44).

Logistic regression analysis was employed to ascertain the factors influencing college students’ awareness of prepackaged nutrition labels. Some of our findings align with existing domestic and international research, while the impact of certain independent variables remains contentious. Variations in the use of nutrition labels were observed between students who have taken nutrition courses and those who have not, which is consistent with a research (27). However, no correlation was found between university students’ gender and label awareness level, inconsistent with one study but consistent with others (45–47). This could be attributed to the gender ratio of the study or other relevant factors. The frequency of using and trust in nutrition labels positively influenced the awareness level, in line with the favorable factors associated with nutrition label application, such as a high valuation of the necessity of nutrition labels and attention to nutrition knowledge (48). It’s concerning that there were differences in nutrition labeling awareness among people with different nutrition labeling habits, but questions about basic nutrition labeling were still the topics with the highest error rates. On the one hand, we believe that this also reflects the poor quality of current nutrition education in China. In the future nutrition education reforms, teachers should consider how to increase student engagement in and out of the classroom so that students can master the knowledge and gradually transition to applying it in their daily lives (49). On the other hand, although college students’ preference for healthy foods seems to correlate positively with their food knowledge, nutrition labeling positively impacts their dietary intake (50, 51). However, college students often do not adhere to the recommended nutrient intake (52). In summary, targeted interventions should be implemented for college students’ nutrition skills and literacy, and the popularity of food nutrition labels should be enhanced (53).

In this investigation, it was observed that 37% of the students still found the nutrition label too complex to comprehend, and 17% of the students expressed mistrust in the content of the nutrition label. Zhiheng Hong et al. (54) also discovered that consumers’ proficiency in reading, comprehending, and correctly utilizing the nutrition label was subpar, highlighting the pressing need to enhance the legibility and credibility of the nutrition label. This situation may be attributed to two primary factors: Firstly, China regulations for prepackaged nutrition labels have only been in effect for a decade, and the associated terminology is not standardized, rendering it challenging for consumers to grasp (55). Secondly, the penetration and accuracy of Chinese food labels are low, with some food labels failing to provide complete, relevant information (56). Furthermore, stricter label management and clearer label presentation can contribute to an improved intake of certain nutrients associated with disease risk and a reduced risk of mortality (57, 58). Consequently, we contend that China should reinforce the evaluation and implementation of pertinent standards and enhance the visual appeal of labels and the communication of label content.

With the continued advancement of society, various countries, such as Sweden and Thailand, have implemented innovative food labeling methods (59, 60). Current nutrition labels predominantly consist of technical information related to food products, which may pose challenges for consumers in understanding the content (61, 62). It is recommended that a more straightforward approach be adopted to inform consumers about the potential health impacts of food consumption (63). For instance, in addition to displaying calorie content, labels could indicate appropriate portion sizes for various demographic groups. To enhance oversight, a governmental system for monitoring health risks associated with prepackaged foods could be implemented. This system could involve assigning a unique QR code to each product, enabling individual tracking of consumer consumption and inclusion of high-risk dietary components in the monitoring process. Furthermore, national dietary guidelines could be linked to trade regulations to promote healthier eating habits on a larger scale. In the future, food labeling will pivot toward personalized, interactive, sustainable labeling, and digital technology applications to meet consumers’ need for nutritional information and to encourage healthy, sustainable, and transparent food consumption. This will provide consumers with more options and decision-making support while guiding the entire food industry toward a healthier and more sustainable path (61).

Although some meaningful conclusions were obtained from this study, several limitations remain. First, the study’s cross-sectional design limited its ability to determine the causal relationship between the factors and college students’ awareness of nutrition labels. Besides, the data from this study came from only five universities in Shanghai, China, which may limit the generalizability and applicability of the findings to other college student populations. To enhance the validity and generalizability of the results, it is recommended that this research be replicated with expanded and heterogeneous populations. Lastly, although this study focused on nutrition education programs, label usage, and college students’ awareness of nutrition labels, it did not assess the actual application or integration of these education programs and nutrition labels in their daily lives. Future research could further explore college students’ actual experiences with nutrition education programs and label usage, and analyze their impact on nutrition label awareness.

## 5 Conclusion

After 10 years of implementing nutrition labels, college students in China, have shown improved awareness of the labels. However, their understanding of the nutrition labels on prepackaged foods remains generally low, and most students still lack basic nutrition knowledge. Surprisingly, the study found that students who took a nutrition course did not exhibit a higher awareness of nutrition labels and nutrition education programs were found to be ineffective in enhancing consumer knowledge of prepackaged nutrition labeling. Furthermore, students who acquired nutrition knowledge from different sources displayed significant differences in their awareness of nutrition labels. Many students still cast doubt on the content of nutrition labels. Hence, there is a need to enhance relevant regulations and policies, strengthen label auditing, improve label credibility, enhance the quality of nutrition education courses, and accelerate the use of digital labels. These efforts aim to promote the correct understanding and utilization of nutritional labeling among university students, with the goal of decreasing the prevalence of chronic diseases. At the same time, this study provides insights for developing countries to optimize the legal system of nutrition labeling and to implement nutrition education and corresponding health interventions.

## Data Availability

The original contributions presented in the study are included in the article/Supplementary material, further inquiries can be directed to the corresponding author.
