# Supplementary material for: Awareness and associated factors of prepackaged food nutrition labels by college students in China: a cross-sectional study
Source: Front Public Health. 2025 Jun 26;13:1577633. doi: 10.3389/fpubh.2025.1577633 (PMC12241032; doi:10.3389/fpubh.2025.1577633)
Supplement: Supplementary file 1 [file Table_1.docx]

**
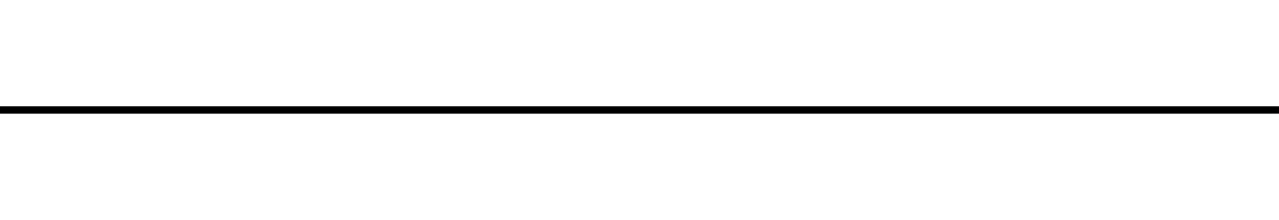
**2024 X Awareness of Prepackaged Nutrition Labeling among College Students Study

**2024 X Awareness of Prepackaged Nutrition Labeling among College Students Study**

**Informed Consent**

1. **Research background**

"（X Awareness of Prepackaged Nutrition Labeling among College Students Study (SCAPLS) was initiated by the Department of X. To investigate the awareness and influencing factors of prepackaged food nutrition labels among college students in X.

1. **Research risk**

This survey is in the form of a self-administered questionnaire, which will have minimal impact on your physical and mental health, the risk is extremely low, and the personal views of the respondents will not affect your life and work.

1. **Privacy issue**

If you decide to participate in this survey, all your personal information involved in the survey will be kept strictly confidential, and we will strictly abide by the relevant provisions of the Statistics Law and will never disclose any of your personal information. All surveyors are required to keep your identity information confidential. All information about you will only be used for academic research and will never be used for other purposes. If the results of the research are to be published, any information that may reveal your identity will be withheld.

1. **Contact information**

If you have questions related to this survey, or if you experience any discomfort during the survey process, or would like to know more about the rights and interests of the participants in this survey, you may call the Department of X.

1. **Informed consent signature**

I have read this informed consent form and the investigator has explained the purpose, content, benefits and risks of this investigation to me in detail and answered all the questions I have asked, I have understood this investigation and I am voluntarily participating in this study.

**Signature of study participant**：

Date：

## Questionnaire No: Date of investigation:

**A. Basic Information**

A1.Gender: □1.Male □2.Female

A2.Weight: (kg)

A3.Height: (cm)

A4. Major

☐1. Business ☐2. Law ☐3. Medical

☐4. Management ☐5. Engineering ☐6. Education

☐7. Languages ☐8. IT

☐9. Art design

A5. Grade: ☐1. Freshman ☐2. Sophomore ☐3. Junior ☐3. Senior

A6. Have you ever studied nutrition course?

☐1. Yes

☐2. No

1. **Use of prepackaged food nutrition labels**

B1. Do you use nutrition labels when you buy food?

☐1. Never

☐2. Sometimes

☐3. Usual

B2. What information do you focus on when you use food labeling?

☐1. Nutritional value table ☐2. Ingredients list

☐3. Shelf life ☐4. Allergen

☐5. Date of production ☐6. Others

B3. What influences you to use nutrition labels?

☐1. Too complicated to read

☐2. Don't believe in nutritional labeling

☐3. Takes a lot of time to read

☐4. Don't care about the information

☐5. Others

B4. What media channels do you often use to obtain nutrition-related knowledge?

☐1. Lecture ☐2. Poster ☐3. Newspaper

☐4. Online Game ☐5. Web video ☐6. Television ☐7. Others

B5. How much do you trust food nutrition labels?

☐1. Very distrustful

☐2. Distrust

☐3. Moderate trust

☐4. Trust

☐5. Very trusting

B6. Do you think that the presence of nutrition labels is necessary in your daily food purchases?

☐1. Necessary

☐2. Normal

☐3. Unnecessary

1. **Prepackaged food nutrition label knowledge**

C1. What are the main things that vegetables provide you with?

☐1. Protein

☐2. Energy

☐3. Carbohydrates

☐4. Vitamins, minerals

C2. What is the recommended daily salt content in X?

☐1. > 5g

☐2. < 5g

☐3. = 5g

C3. What does "Recommended National Dietary Nutrient Intakes" mean?

☐1. The average of the nutrient requirements for a given age, sex, and physiologic group.

☐2. An intake level that meets the needs of the majority of individuals of a particular sex, age and physiological status group

☐3. A reference value for the average daily reference intake of a nutrient in the diet.

☐4. The maximum amount of a nutrient that can be consumed on an average daily basis.

C4. What does "sugar-free" mean in food labeling?

☐1. No sugar content

☐2. Sugar content ≤ 0.5g in 100g

☐3. Sugar content > 0.5g in 100g

☐4. Sugar content < 0.3g in 100g

C5. Which of the following is a mandatory mark for nutrition labeling of food?

☐1. Nutrition Facts Table

☐2. Nutrition Claims

☐3. Nutrient Function Claims

C6. What is the NRV% meaning of the following images?


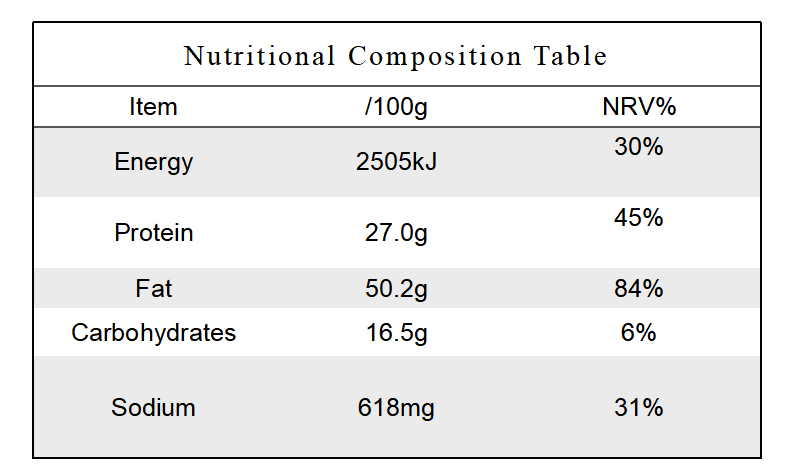


☐1. 45% of the protein in 100g of food can be absorbed by the body

☐2. 100g of food contains 30% of the body's daily energy requirements

☐3. 84% fat in 100g of food

☐4. 6% of carbohydrates in 100g of food meets the body's daily needs

C7. Dietary carbohydrates should account for energy?

☐1. 30%

☐2. 40%

☐3. 70%

☐4. 60%

C8. Daily dietary fat should not provide more than 30% of total energy?

☐1. Yes

☐2. No

C9. Are the following foods suitable for children?


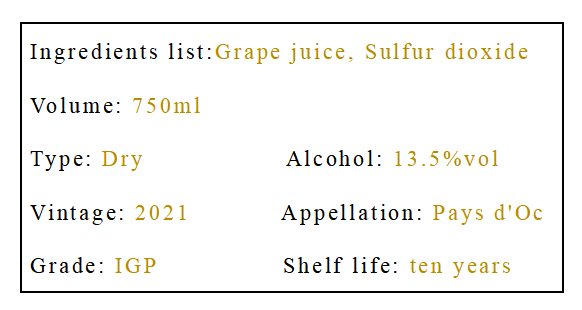


☐1. Yes

☐2. No

C10. Core nutrients for nutrition labeling include: protein, fat, carbohydrates and sodium?

☐1.Yes

☐2. No

C11. Does food labeling refer to the words, graphics, symbols and descriptions on food packaging?

☐1.Yes

☐2. No

Thank you for completing this questionnaire!
